# The Chinese medicine formula HB01 reduces choroidal neovascularization by regulating the expression of vascular endothelial growth factor

**DOI:** 10.1186/1479-5876-10-118

**Published:** 2012-06-07

**Authors:** Ming Jin, Youhua Zhang, Lin Pan, Renhui Dou, Robert B Nussenblatt, Lai Wei

**Affiliations:** 1 Department of Ophthalmology, China-Japan Friendship Hospital, Beijing University of Chinese Medicine, Beijing, China; 2 Laboratory of Immunology, National Eye Institute, National Institutes of Health, Bethesda, MD, USA; 3 National Center for Complementary and Alternative Medicine, National Institutes of Health, Bethesda, MD, USA

**Keywords:** Choroidal neovascularization, Traditional Chinese medicine, VEGF

## Abstract

**Background:**

Choroidal neovascularization (CNV) remains the leading cause of newly acquired blindness in the developed world. Currently anti-vascular endothelial growth factor (VEGF) therapies are broadly used to treat neovascular ocular disorders. Here we demonstrate the effect of a traditional Chinese medicine formula, HB01, on CNV.

**Methods:**

A rat model of laser-induced CNV was used to investigate the effect of HB01 *in vivo*. The CNV lesions in the eye were evaluated using fundus fluorescein angiography and visualized/quantified using confocal microscopy. Expression of VEGF in the choroidal and retinal tissues was measured using quantitative real-time PCR and immunohistochemistry.

**Results:**

We demonstrated that a traditional Chinese Medicine formula, named HB01, significantly reduced neovascularization in a rat CNV model. The effect of HB01 on CNV was comparable to the intravitreal injection of bevacizumab (Avastin). Our results also suggested that HB01 may reduce CNV partially through inhibiting the expression of VEGF.

**Conclusions:**

These data support HB01 as an alternative therapy for ocular neovascular disorders.

## Background

Many ocular diseases, such as the wet or exudative/neovascular age-related macular degeneration (AMD), myopia and diabetic retinopathy, involve in choroidal neovascularization (CNV), which remains the leading cause of newly acquired blindness in the developed world [1]. In neovascular AMD, blood vessels compromise Bruch’s membrane from the choroid and grow into the space beneath the retinal pigment epithelium (RPE), or within the subretinal space between RPE and photoreceptor. The loss of visual acuity is caused by serum or blood leaked from the proliferating blood vessels underneath and within the retina. A subretinal fibrovascular scar eventually develops if the CNV is left untreated, leading to a permanent loss of vision [2]. On the other hand, in proliferative diabetic retinopathy, neovascularization originates in the retinal vessels and appears on the surface of the retina. These leaky blood vessels can lead to hemorrhages and fluid in the retinal tissue and vitreous, which compromises vision [3].

Several treatments for CNV are currently available, with variable efficacy on disease progress, including local corticosteroids, submacular surgical removal of CNV, laser photocoagulation, and photodynamic therapies [4,5]. More recently, the development of drugs directly suppressing the growth and development of blood vessels provides new outlooks for effectively controlling CNV. A number of large randomized clinical trials suggest that vascular endothelial growth factor (VEGF) is a central player in pathogenesis of CNV and inhibiting its function locally in the eye is sufficient to cause a decrease in angiogenesis that leads to a relief of symptoms in wet AMD and other neovascular ocular diseases. Among all anti-VEGF therapeutic agents, the anti-VEGF antibody drugs ranibizumab (Lucentis, Genentech) and bevacizumab (Avastin, Genentech) are most broadly used to treat AMD with similar efficacy and side effects [6,7].

Although current therapeutic options to treat CNV are effective for the majority of patients, we have been looking for more successful and definitive alternatives with improved efficacy, reduced cost, and less complications. A rich literature including ancient Chinese medical records and modern Chinese studies in the past decades demonstrates some success of traditional Chinese medicine treating neovascular ocular diseases such as wet AMD [8-10]. We have previously shown that a traditional Chinese medicine formula, HB01, can reduce CNV and hemorrhage in the eyes as well as improve the visual acuity in patients with neovascular AMD, pathological myopia, or central exudative chorioretinitis [11]. HB01 is the water extract of six herbs including *Astragalus membranaceusAngelica sinensisPanax NotoginsengPollen TyphaeBulbus Fritillariae ThunbergiiCitrus reticulata*, in the ratio of 1:1:1:1:1:1 (9 g each). The major functional component of HB01, *Astragalus membranaceus*, has been used for thousands of years in Asia to prevent cold and respiratory infections. It has both anti-bacterial and anti-inflammatory effects [12]. While *Angelica sinensisPanax Notoginseng*, and *Pollen Typhae* can stop bleeding [13-15], *Citrus reticulata* has been used to relieve congestion in the lung [16]. Therefore, HB01 has both anti-inflammatory and anti-hemorrhage effects. Our current study is to investigate whether HB01 can limit the formation of CNV and the molecular mechanism underlying its effect in a laser-induced CNV rat model.

## Methods

### HB01

HB01 is comprised of six herbs, *Astragalus membranaceus*, *Angelica sinensis*, *Panax Notoginseng*, *Pollen Typhae*, *Bulbus Fritillariae Thunbergii*, *Citrus reticulate*, in the ratio of 1:1:1:1:1:1 (9 g each), respectively. The water extract (total of 500 ml per day) of mixture of all herbs was prepared and packed by the department of Pharmaceutical Sciences, China-Japan Friendship Hospital, Beijing University of Chinese Medicine (Beijing, China) using standardized procedure.

### Laser-induced CNV in rats

Eight week old male Brown Norway rats weighing between 200 and 250 g were purchased from Vital River Inc. (Beijing, China) and maintained in accordance with the Institutional Animal Care and Use Committee procedures and guidelines, as well as ARVO guidelines on the use of Animals in Ophthalmic and Vision Research. The CNV was induced as previously described [17,18]. Briefly, rats were anesthetized by intraperitoneal injection of ketamine (70 mg/kg) and xylazine (7 mg/kg). The left pupil was dilated with 1 drop of 1% Tropicamide (Beijing Double-Crane Pharmaceutical Business Co., Beijing, China). Then the left eye received 10–12 laser burns between retinal vessels around the optic nerve head (360 mW, 50 ms, 0.05 mm spot size) using an ARAMIS II Argo Laser (Quantel Medical, France). The rupture of Bruch’s membrane was confirmed in all laser lesions. Animals were divided into 4 groups and treated as following: 1) *Control* group did not received laser or drug treatment, and was given 2 ml/0.2 kg PBS once daily by oral gavage starting on Day 0 for 4 weeks; 2) *CNV* group received laser on Day 7 but not drug treatment, and was given 2 ml/0.2 kg PBS once daily by oral gavage starting on Day 0 for 4 weeks; 3) *CNV with bevacizumab* group received laser on Day 7 and one intravitreal injection (25 ug in 1 ul solution, on Day 14) of bevacizumab (Roche, USA); 4) *CNV with HB01* group received laser on Day 7 and HB01 (once daily by oral gavage, 2 ml/0.2 kg, starting on Day 0 for 4 weeks). The dose of HB01 used in rats was calculated according to the one used in our previous study in AMD patients (500 ml/50 kg/day) [11].

### Fundus fluorescein angiography

CNV lesions were evaluated by fundus fluorescein angiography as previously described [18]. On Day 7, 14, 21, and 28 after laser photocoagulation, the CNV lesions were visualized using a digital fundus camera (Topcon, Japan). The mean area of CNV was calculated using Image-Pro Plus 6.0 (Kodak, USA) by two independent ophthalmologists blinded to the experimental design.

### Visualizing and quantifying CNV

CNV was visualized and quantified as previously described [18]. Briefly, rats were anesthetized, killed with 100% CO2, and perfused through the left ventricle injection of 1 ml PBS, followed by perfusion of lactated Ringer’s solution (Abbott Laboratories, USA) containing 10% gelatin with 5 mg/ml FITC-Dextran (Sigma, USA). The eyes were removed and fixed in 10% formalin for 1 hour. The sclera-choroid-RPE complex was flat-mounted after removing the cornea, lens and the neurosensory retina. Flat mounts were visualized using a confocal microscope (Zeiss LSM510). Images of the neovascular lesions were captured using a digital camera (OlympusBX-51) and analyzed using Image-Pro Plus 6.0 (Kodak, USA). A laser spot with green vessels were scored CNV positive, while a laser spot lacking green vessels were scored CNV-negative.

### Histopathology and Immunohistochemistry

On Day 28, the eyes were enucleated and fixed in 4% paraformaldehyde overnight. The posterior eye cup was embedded in paraffin and serial sections of 4 μm thick were cut and used for either H&E staining or immunostaining. To measure the thickness of CNV lesions, the sections with the largest areas of the CNV lesions were selected. The maximum thickness of the selected CNV lesion centers were recorded by a masked pathologist. The rabbit polyclonal anti-VEGF antibody and rabbit anti-human IgG (Wuhan Boster Bio-Engineering LLC, China) were used in immunohistochemistry analysis.

### RNA isolation and quantitative real-time PCR

The retinal and choroidal tissue was dissected from the rat eyes on Day 28 and RNA was extracted using TRIzol reagent (Invitrogen, USA). The reverse transcription was performed using PrimeScript RT reagent Kit (TaKaRa Bio Inc, Japan) according to the protocol manufacture provided. Real-time PCR was performed with SYBR Green PCR Master Mix on the ABI PRISM 7500 Sequence Detection System (Applied Biosystems, USA). The relative expression of *Vegfa* was normalized to *Actb*. The following primer sets were used:

Actb-F: CCTGTATGCCTCTGGTCGTA, Actb-R: TGAGCTATGAAGGCGACGTTACCA; Vegfa-F: CTCACCAAAGCCAGCACATAGGAGAG, Vegfa-R: TCTGCGGATCTTGGAC-AAACAAATGC.

### Statistical analysis

All data were analyzed using GraphPad Prism 5.0 (GraphPad Software, USA). Statistical significance was determined by Mann–Whitney test or Chi-square test as indicated in the figure legend. A P value less than 0.05 is considered statistically significant.

## Results

### Prevention of CNV formation by HB01

**Figure 1:**
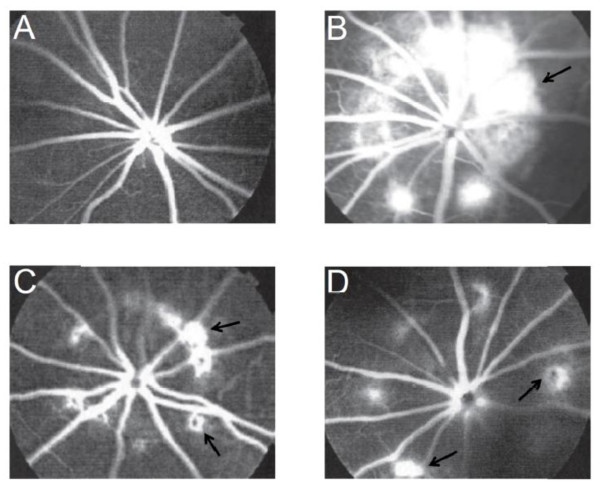
** Fundus fluorescein angiography.** (A) Fundus fluorescein angiography of a representative rat in the Control group that did not received laser or drug treatment. (B) Fundus fluorescein angiography of a representative rat in the CNV group that received laser on Day 7 but not drug treatment. (C) Fundus fluorescein angiography of a representative rat in the CNV with bevacizumab group that received laser on Day 7 and one intravitreal injection of bevacizumab on Day 14. (D) Fundus fluorescein angiography of a representative rat in the CNV with HB01 group received laser on Day 7 and HB01 treatment for four weeks. Arrows marked lesions.

**Figure 2:**
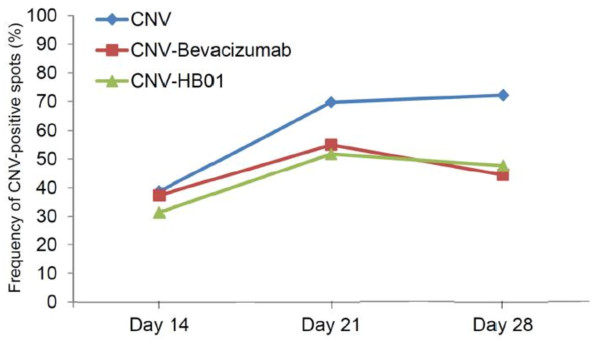
** The frequency of CNV-positive spots.** The CNV lesions were evaluated on Day 14, Day 21, and Day 28. The percentage of CNV positive spots was calculated as the ratio of CNV positive spots (in all eyes within a group of rats) and all CNV lesions.

**Figure 3:**
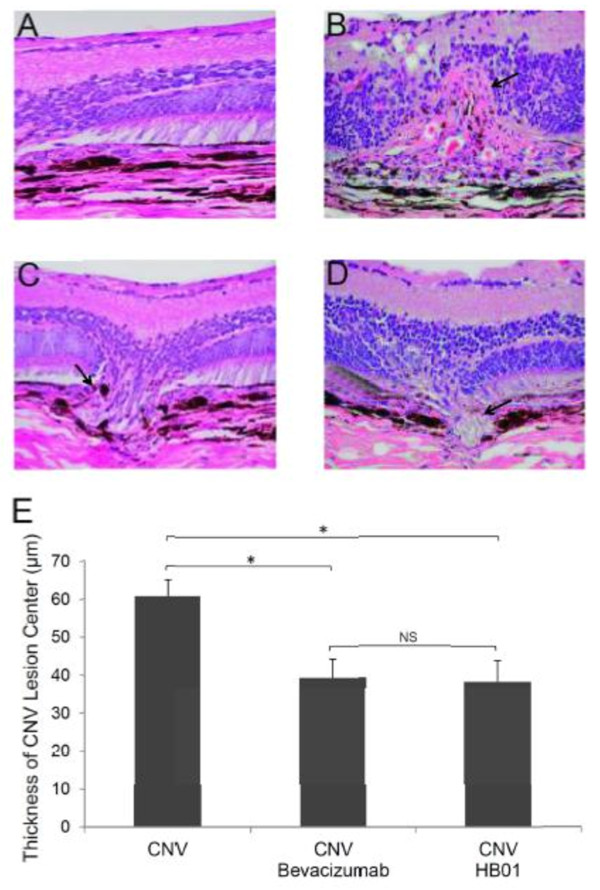
**Histologic analysis of CNV lesions in rat eyes**. Paraffin-embedded cross sections through CNV lesions were examined using Hematoxylin/eosin (H&E) staining for eyes from the Control (A), CNV (B), CNV with bevacizumab (C), and CNV with HB01 (D) groups on Day 28. The thickness of the retinal lesion centers was summarized in (E). *P < 0.01, Mann Whitney test; NS, not significant. Arrows marked CNV lesions.

To determine the effect of HB01 on laser induced CNV, we first carried out fundus fluorescein angiography. Rats were divided into four groups (12 rats in each group) as indicated in the Methods Section. The CNV was evaluated on Day 14, 21, and 28 after laser induction of CNV spots on Day 7. As shown in Figure 1, robust choroidal neovascular lesions formed in lasered, PBS treated rats (Figure 1B) on Day 28, while rats treated with both bevacizumab (Figure 1C) and HB01 (Figure 1D) appeared to have fewer and smaller lesions. To quantify the incidence of CNV formation, we calculated the percentage of CNV-positive spots among all lesion spots. On Day 14, the numbers of CNV-positive spots for all three groups receiving laser were not different (31-38%) (Figure 2). However, interestingly, both bevacizumab and HB01 significantly reduced the numbers of CNV-positive spots in the rat eyes on Day 21 and 28 (Figure 2). No difference was found in regression of CNV between bevacizumab and HB01 groups. In addition, we performed histologic analysis of paraffin-embedded cross section of the rat retinal tissues. As shown in Figure 3, laser induced damage of retinal and choroidal structure and broad neovascularization in untreated rats (Figure 3B), as compared to the normal eyes (Figure 3A). Both bevacizumab (Figure 3C) and HB01 (Figure 3D) reduced neovascularization and partially restored the normal retinal and choroidal structure. In addition, both treatments significantly reduced the thickness of retinal lesions caused by CNV (Figure 3E). These results indicate that the Chinese medicine formula HB01 may induce regression of laser-induced CNV, similar to the effect of intravitreal injection of bevacizumab.

**Figure 4:**
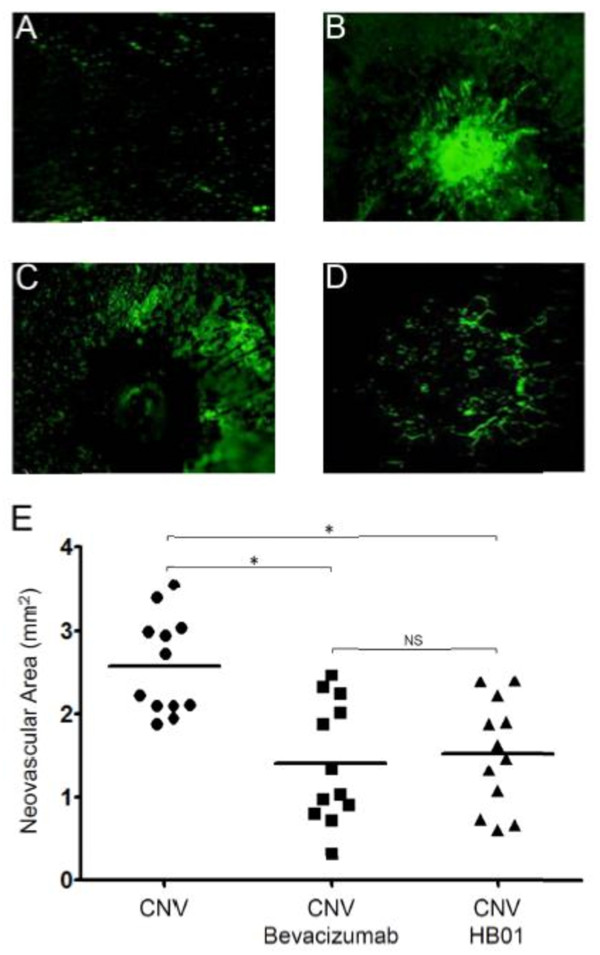
**Confocal microscopy of CNV lesions**. The fluorescent images of CNV in RPE-choroid-sclera flat mounts from the Control (A), CNV (B), CNV with bevacizumab (C), and CNV with HB01 (D) groups were shown. The vasculature was labeled with FITC-dextran via cardiac perfusion on Day 28. The total sizes of neovascular areas for eyes from CNV, CNV with bevacizumab, and CNV with HB01 were summarized in (E). *P < 0.01, Mann Whitney test; NS, not significant.

To further determine whether HB01 can decrease neovascularization, we measured the size of CNV in the images of FITC-dextran labeled RPE-choroid-sclera flat mounts. On Day 28, an area of hyperfluorescence with a diameter approximately equal to that of the laser spot was found only in the eyes received laser but no drug treatment (Figure 4B), while both bevacizumab (Figure 4C and 4E) and HB01 (Figure 4D and significantly reduced the size of laser4E) significantly reduced the size of laser-induced CNV formation. Taken together, our results suggest a significant effect of the Chinese medicine formula HB01 on reduction of CNV *in vivo*.

### Expression of VEGF regulated by HB01

**Figure 5:**
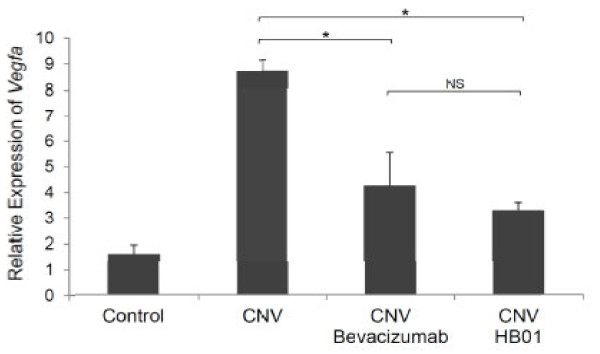
** Expression of*Vegfa*mRNA**. Shown is the relative expression of *Vefga* mRNA on Day 28 in choroidal and retinal tissues (n = 12 for each group). Values are mean ± SD. *P < 0.01, Mann Whitney test; NS, not significant.

**Figure 6:**
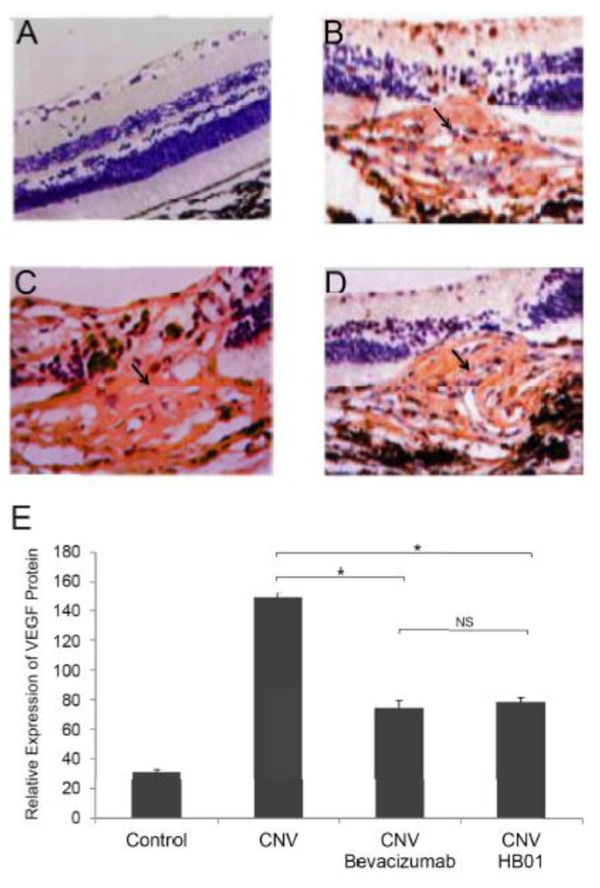
** Expression of VEGF protein**. Shown is the immunostaining of VEGF in the CNV lesions on Day 28 in eyes from the Control (A), CNV (B), CNV with bevacizumab (C), and CNV with HB01 (D) groups. The relative expression of VEGF protein was summarized in (E). *P < 0.01, Mann Whitney test; NS, not significant. Arrows marked CNV lesions.

To investigate the potential molecular mechanism underlying the effect of HB01 on CNV formation, we performed quantitative Real-time PCR and immunohistochemistry analysis to evaluate the expression of VEGF, the potent angiogenic factor previously shown to be responsible for promoting CNV. Consistent with previous studies [19], intravitreal injection of bevacizumab significantly reduced expression of *Vegfa* mRNA (Figure 5) and its protein (Figure 6C and 6E) in lasered eyes, as compared to the one received no drug treatment (Figure 5 and Figure 6B and 6E). Intriguingly, oral administration of HB01 also resulted in a significantly reduction of *Vegfa* mRNA (Figure 5) and its protein (Figure 6D and 6E) in lasered eyes, as compared to the one received no drug treatment (Figure 5 and Figure 6B and 6E). These results suggest that HB01 may function to reduce CNV by controlling the expression of VEGF.

## Discussion

Laser induced CNV model has been established in both non-human primates and rodents [20-22]. It shows remarkable similarity to the pathology found in many neovascular ocular disorders such as wet AMD. It has been extensively used to evaluate the efficacy of anti-CNV therapies [1]. In our study, we demonstrated that a traditional Chinese Medicine formula, named HB01, significantly reduced neovascularization in a rat CNV model. The effect of HB01 on CNV was comparable to the intravitreal injection of bevacizumab (Avastin). Our results also suggested that HB01 may reduce CNV partially through inhibiting the expression of VEGF. To our knowledge, this is the first attempt to understand whether and how traditional Chinese medicine can reduce CNV in a rodent model.

Current therapeutic strategies for CNV often involve in invasive approaches such as intravitreal injection or surgery. However, none of these treatments fully restore the lost vision in most patients and they can result in complications leading to further vision loss [23,24]. The oral administration of traditional Chinese herbal medicine could provide an alternative but effective approach for CNV therapy. Often time, thousands of years of clinical experiments have optimized formulas in Chinese medicine. Therefore, it will be reasonable to expect that most of the classical formulas work to some extent as indicated in the ancient literature. The formula HB01 exemplifies the expectation and shows its effectiveness in reducing CNV in a small randomized clinical trial [11]. However, our current and further studies aim to elucidate the molecular mechanism by which HB01 reduces CNV. In addition, a therapy combining both HB01 and anti-VEGF treatment will be especially interesting, which could improve the efficacy and safety as well as limit the complications associate with the therapy.

Currently, there is a great debate about use of Chinese herbal medicine as a sole treatment for diseases, considering its potential placebo-only effect [25]. Moreover, producing an herbal formula with constant active ingredients remains a great challenge [26]. In addition, the laser-induced rat CNV model involves a wound healing process that is potentially different from the pathogenesis of wet AMD. It is necessary to investigate the effect of HB01 on other AMD animal models. Moreover, further study is warranted to characterize chemical composition of HB01 and its drug targets *in vitro* and *in vivo*.

## Conclusions

Our results demonstrate that HB01 reduced CNV in a rat model of laser-induced CNV, probably by inhibiting the expression of VEGF. This data support HB01 as an alternative therapy for ocular neovascular disorders.

## Competing interests

MJ has a patent (CN102058845A) on the HB01 used in this study.

## Authors’ contributions

MJ and YZ designed the study. YZ, LP, and RD carried out experiments. MJ and LW performed statistical analysis. MJ, RBN, and LW participated in the design of study, interpretation of results, and drafted the manuscript. All authors read and approved the final manuscript.
